# Reconfigurable MRI coil technology can substantially reduce RF heating of deep brain stimulation implants: First in-vitro study of RF heating reduction in bilateral DBS leads at 1.5 T

**DOI:** 10.1371/journal.pone.0220043

**Published:** 2019-08-07

**Authors:** Laleh Golestanirad, Ehsan Kazemivalipour, Boris Keil, Sean Downs, John Kirsch, Behzad Elahi, Julie Pilitsis, Lawrence L. Wald

**Affiliations:** 1 Department of Biomedical Engineering, McCormick School of Engineering, Northwestern University, Evanston, IL, United States of America; 2 Department of Radiology, Feinberg School of Medicine, Northwestern University, Chicago, IL, United States of America; 3 Department of Electrical and Electronics Engineering, Bilkent University, Ankara, Turkey; 4 Department of Life Science Engineering, Institute of Medical Physics and Radiation Protection, Giessen, Germany; 5 A. A. Martinos Center for Biomedical Imaging, Massachusetts General Hospital, Boston, MA, United States of America; 6 Department of Neurology, Bryan Health, Lincoln, NE, United States of America; 7 Department of Neurosurgery, Albany Medical Center, Albany, NY, United States of America; McLean Hospital, UNITED STATES

## Abstract

Patients with deep brain stimulation (DBS) implants can significantly benefit from magnetic resonance imaging (MRI), however access to MRI is restricted in these patients because of safety concerns due to RF heating of the leads. Recently we introduced a patient-adjustable reconfigurable transmit coil for low-SAR imaging of DBS at 1.5T. A previous simulation study demonstrated a substantial reduction in the local SAR around single DBS leads in 9 unilateral lead models. This work reports the first experimental results of temperature measurement at the tips of bilateral DBS leads with realistic trajectories extracted from postoperative CT images of 10 patients (20 leads in total). A total of 200 measurements were performed to record temperature rise at the tips of the leads during 2 minutes of scanning with the coil rotated to cover all accessible rotation angles. In all patients, we were able to find an optimum coil rotation angle and reduced the heating of both left and right leads to a level below the heating produced by the body coil. An average heat reduction of 65% was achieved for bilateral leads. When considering each lead alone, an average heat reduction of 80% was achieved. Our results suggest that reconfigurable coil technology introduces a promising approach for imaging of patients with DBS implants.

## Introduction

Deep brain stimulation (DBS) is a reversible and adjustable neurostimulation technique in which specific brain structures and circuits are electrically stimulated by means of metallic electrodes connected to an implantable pulse generator (IPG) via subcutaneous insulated wires. The US Food and Drug Administration (FDA) approved DBS as a treatment of essential tremor and Parkinson’s disease (PD) in 1997, dystonia in 2003 [1], obsessive-compulsive disorder (OCD) in 2009 [2], and recently epilepsy in 2018 [3]. Beside this, in recent years DBS has been extensively used in open-label studies to treat Tourette’s Syndrome [4, 5], bipolar disorder [6], Schizophrenia [7], treatment-resistant depression [8, 9], and freezing of gait [10]. The exponential increase in indications of use and application of DBS in neuropsychiatric disorders parallels the large availability and need for MRI. Particularly, MRI has been largely leveraged to understand the underlying mechanisms of action of neuromodulation systems and more recently to guide therapy. The clinical community however, has been cautious in adopting MRI in DBS patients mostly due to concerns about interaction of MRI fields and the implanted device. This includes for example, distortions that affect image quality (e.g., an in-situ device induces susceptibility artifacts), interaction with static magnetic field, loss of device functionality due to interference of gradient fields, but most importantly the potential of thermal injury to patients due to interaction with radiofrequency (RF) fields. Such concerns have led many centers to refrain from performing postoperative MRI on DBS patients mainly to adhere to industry-proposed warnings [11]. In some cases, patients have faced the proposition of explanting their neurostimulator to receive a diagnostic MRI [12]. With the advances in neurostimulation technology, some of the above-mentioned concerns have been mitigated: reduction of ferromagnetic material has reduced the risk of device dislodgment due to static magnetic field [13] and improved device programming has reduced the risk of malfunction due to effect of gradient fields [14]. RF heating however, remains a major issue. The RF safety of MRI in patients with elongated implants has been extensively discussed in the literature [15–23]. Techniques based on parallel transmit pulse tailoring [23–26] and surgical lead management [20, 27] have shown promising results in reducing the temperature rise near implanted wires in phantoms during MRI at 1.5T and 3T, but such techniques have not been implemented in clinical settings yet. Recently we introduced a patient-adjustable reconfigurable MRI coil with promising results in reducing SAR amplification near tips of implanted leads [28, 29]. The coil system consisted of a linearly-polarized rotating birdcage transmitter and a 32-channel close-fit receive array. Such a linearly-polarized transmit coil has a slab-like region of low electric field which can be steered to coincide with the implant trajectory by rotating the coil around patient’s head. This will significantly reduce the coupling of electric fields with implanted wires, which in turn reduces the RF induced currents on the leads and the SAR at the tip. In a simulation study with patient-derived realistic models of 9 unilateral DBS leads we demonstrated that a substantial reduction in the local energy deposition can be achieved using this technique [29]. In practice however, most patients with PD or essential tremor receive bilateral implants as it has been shown more effective at controlling appendicular and midline tremor [30]. Additionally, 52% of patients who originally receive unilateral DBS eventually need bilateral stimulation [31]. Considering the fact that the optimal coil rotation angle to minimize the local SAR is dependent on the specific lead trajectory [29], the question arises as whether or not the rotating coil system can ever be used in patients with bilateral DBS implants. In other words, as the maximum SAR-reduction occurs when the lead is fully contained in the low electric field region of the coil, it is crucial to assess if there exists a rotation angle that reduces the heating of both left and right DBS leads to the level below the heating generated by a conventional quadrature coil.

In this work we report, for the first time, measurement results of temperature rise at the tips of bilateral DBS lead implants with realistic trajectories during MRI at 1.5 T using the rotating coil system. Implant trajectories were extracted from CT images of 10 patients with bilateral DBS leads (20 leads in total). Anthropomorphic head phantoms were constructed and implanted with bilateral lead wires and positioned inside the coil system with temperature probes attached at their tips. For each experiment, the coil was rotated around the head phantom covering the full range of accessible angles and the temperature at the tips of implants was recorded at each rotation angle during 2 minutes of continues RF exposure. A total of 200 measurements were performed (10 patients×16–22 measurements per patient). We found that for all realistic bilateral lead trajectories, there existed an optimum coil position that substantially reduced the heating of both left and right DBS leads compared to the scanner’s body coil. An average heat reduction of 65% was achieved in the case of bilateral leads. In the case of single leads, an average heat reduction of 80% was achievable using the rotating coil system.

This work reports the first experimental results of successful application of the reconfigurable coil technology to reduce heating of bilateral DBS leads.

## Methods

### The reconfigurable coil system

**Fig 1:**
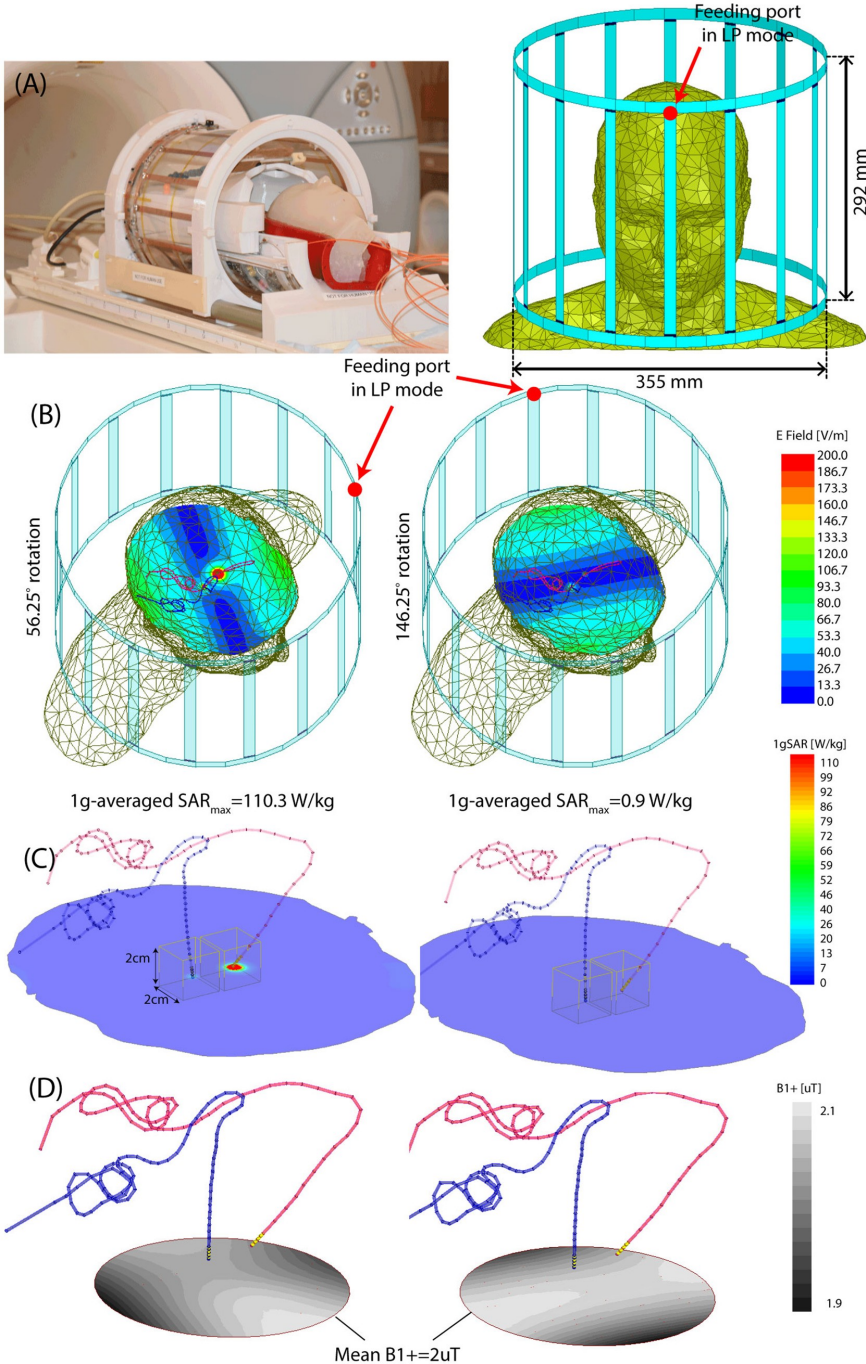
An overview of the reconfigurable MRI coil system. (A) View of constructed prototype and its finite element model. (B) Electromagnetic simulations giving the magnitude of electric field E and maximum of 1g-averaged SAR on an axial plane passing through the center of the head. Simulations are performed with DBS leads of patient ID10 in Fig 3. (C) Maximum 1g-averaged SAR is calculated inside a cubic area surrounding all electrode contacts. (D) The input power of the coil was adjusted to produce the mean B1+ = 2μT on a circular axial plane at the center of the head. The SAR was substantially reduced when the coil was rotated such that the majority of lead trajectory was contained within the region of low electric field.

Details of the theory and construction of the reconfigurable birdcage coil system are given in our previous works [28, 29]. In brief, the coil consists of a 16-rung linearly-polarized low-pass birdcage transmitter and a 32-channel close-fit receive array Fig 1A). Such a linearly-polarized birdcage has a slab-like region of low electric field which can be steered by rotating the coil around patient’s head to coincide with the implant trajectory. Fig 1B shows the distribution of coil’s electric field on a central axial plane for two different coil rotation angles. The details of coil’s model and finite element simulation setup is given elsewhere [28]. To illustrate the orientation of DBS leads with respect to the coil’s electric field, models of bilateral leads were constructed from CT images of a representative patient (ID10). Image segmentation and 3D model construction are described in our previous work [20]. As it can be observed, by rotating the coil around patient’s head and positioning it at an optimal angle, a substantial portion of the lead trajectory can be contained within the low E field region. This reduces the coupling of the electric fields and conductive wires which in turn reduces the SAR at the implant’s tip. We calculated the maximum of 1g-averaged SAR in two cubic areas of 2cm×2cm×2cm containing all four electrode contacts of each lead. Fig 1C reports the maximum SAR value at two different rotation angles. It is important to note however, that the SAR-reduction performance of the coil and the optimum rotation angle that minimizes the SAR at the tip of each implant is dependent on the lead trajectory [29]. This is in line with previous studies that have emphasized on the importance of the relationship between implant’s geometry, and the phase and orientation of incident electric field of MRI transmit coils [25, 32–37]. For each simulation, the B_1_^+^ field was calculated as B_1_^+^ = 0.5(B_1x_+jB_1y_) [38] and its value was recorded on an axial circular plane (diameter = 5cm) passing through the center of the head approximately 1 cm below the tips of electrodes (Fig 1D). The input power of the coil was adjusted at each rotation angle to generated the same spatial mean B_1_^+^ = 2μT over this central plane.

**Fig 2:**
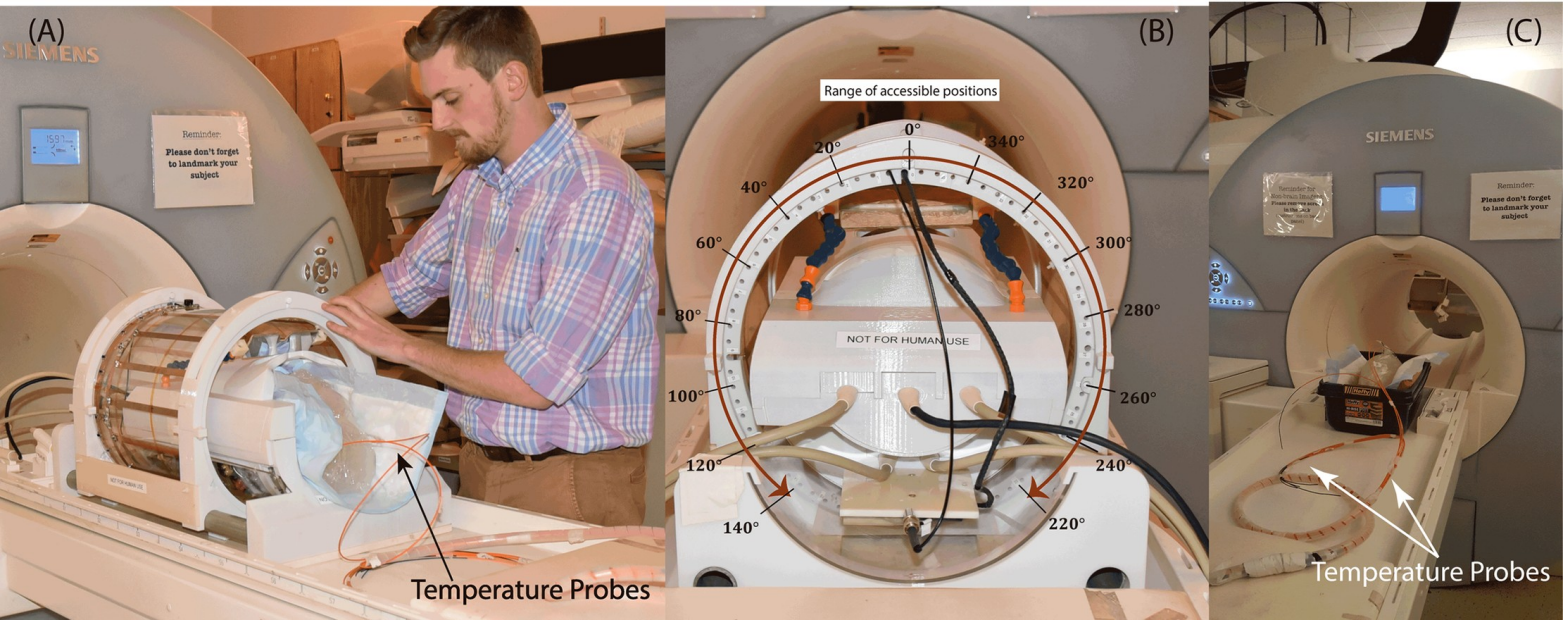
(A-B) The positioning of the rotating coil system on the patient’s table and the range of accessible rotating angles. (C) Phantom scanned with the body coil for comparison. The body coil generated the same level of whole-head SAR as the rotating coil.

Fig *2*B and *2*C shows the constructed coil prototype on the patient’s table and the view from back of the coil. The mechanical housing allows the transmitter to rotate smoothly around the receive array and be locked in pre-marked positions which are 5° apart. When positioned at the magnet’s iso-center, the coil can be accessed and rotated from the back of the bore, eliminating the need to take the patient out for adjustments. Due to mechanical restrictions imposed by positioning of the cables however, the coil cannot rotate in a full circle. The range of accessible angles (0°-140° and 220°-360°) are annotated in Fig *2*.

### DBS leads and head phantoms

**Fig 3:**
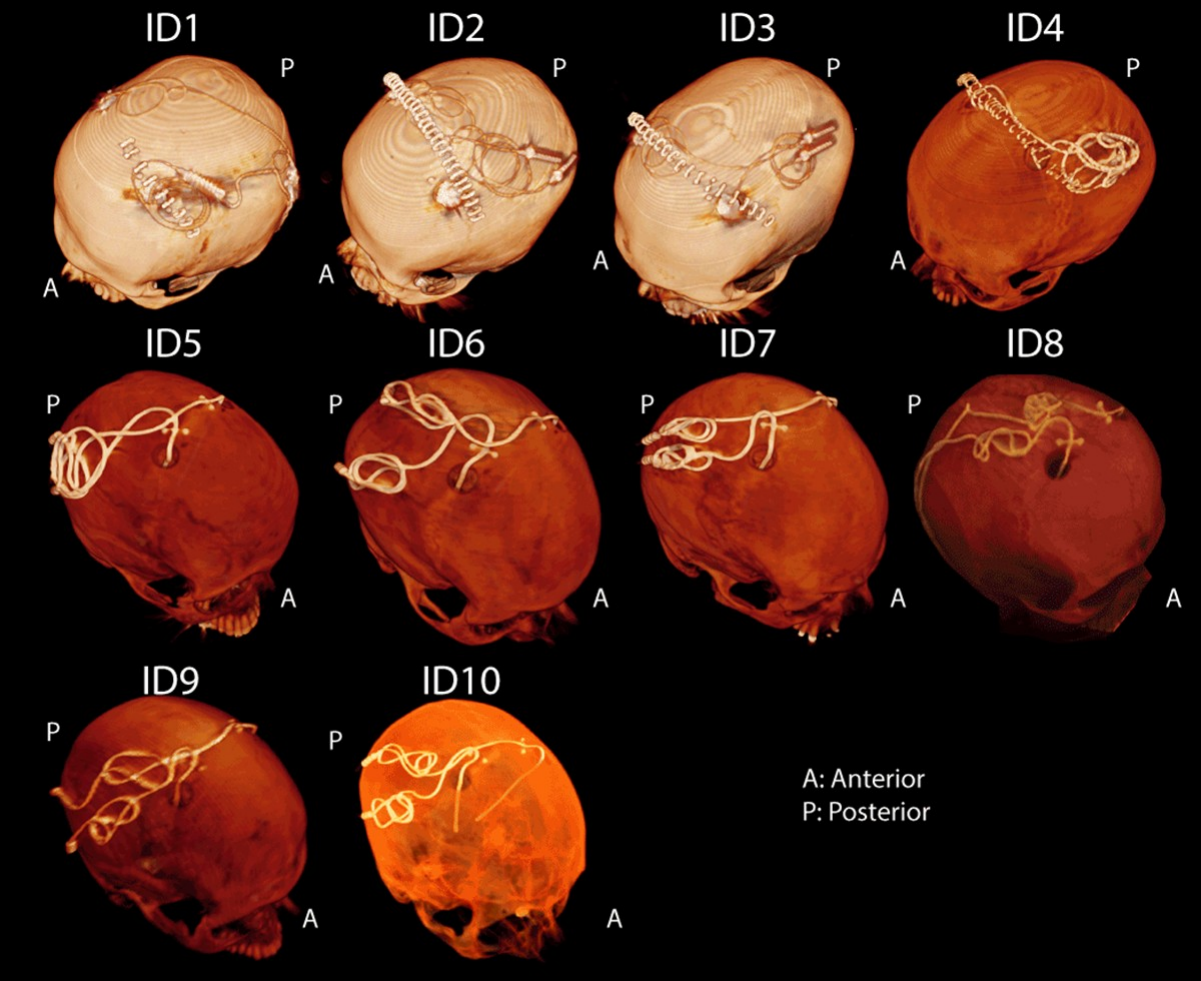
CT images of 10 patients operated for bilateral DBS implantation. For patients 1–4, the IPG was planned to be implanted in the left pectoral pocket. Note that end segments of both leads are routed toward the left side of the head in these patients. In these cases, the left lead was labeled as ipsilateral and right lead was labeled contralateral when representing the temperature results in Figs 6 and 7. For patients 5–10 the IPG was planned to be implanted in the right pectoral region, thus in these patients the right lead was labeled ipsilateral and left lead was labeled as contralateral.

**Fig 4:**
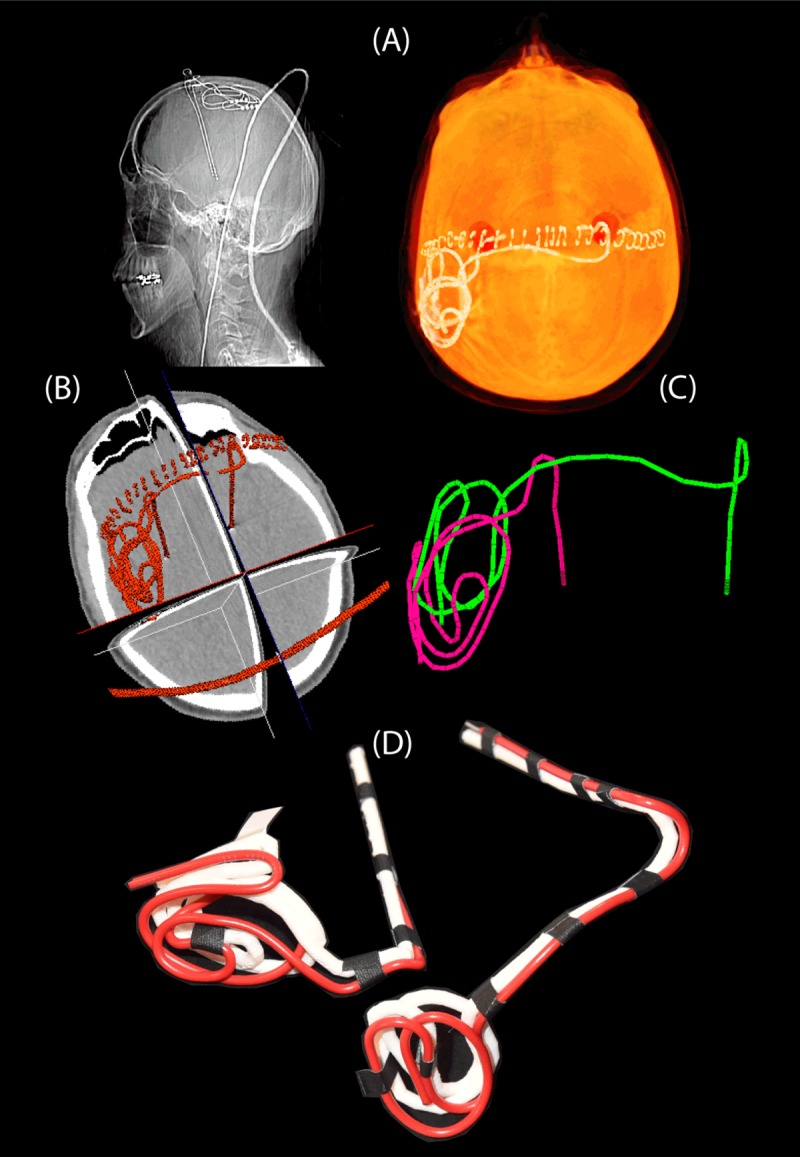
Steps of image segmentation (A-B), 3D model construction (C), and 3D printed plastic guides (D).

Postoperative CT images of ten patients who had undergone surgery for bilateral DBS implantation was used to extract lead trajectories (Fig *3*). The secondary use of imaging data for modeling and simulation was approved by the ethics review board of Massachusetts General Hospital. A total of 20 leads were constructed for measurements. Amira software (Thermo Fisher Scientific, Waltham MA) was used for image segmentation and construction of the preliminary 3D surface of the leads. First, a thresholding mask was applied to select the hyper dense DBS lead from CT images using Amira’s segmentation module (Fig 4A and 4B). Threshold values were selected manually on a case-by-case basis such that the resulting mask produced a smooth continuous 3D surface. 3D lead surfaces were exported to a CAD tool (Rhino3D, Robert McNeal and Associates, Seattle, WA) in which lead trajectory lines were manually extracted, thickened (4mm diameter), and prepared for 3D printing (Fig 4C). Lead guides were then 3D printed out of polycarbonate plastic using a Fortus 360mc 3D printer (Stratasys, Eden Prairie, MN, USA). Two pieces of insulated wire (Ga 14, solid core, McMaster-Carr Elmhurst, IL) each 40 cm long with 1cm exposed tip were shaped around 3D printed guides to follow the left and right lead trajectories (Fig 4D). Wires were rigid enough to maintain their shape once they were routed around the plastic guides and were detached from the guide before being implanted into the head phantom.

**Fig 5:**
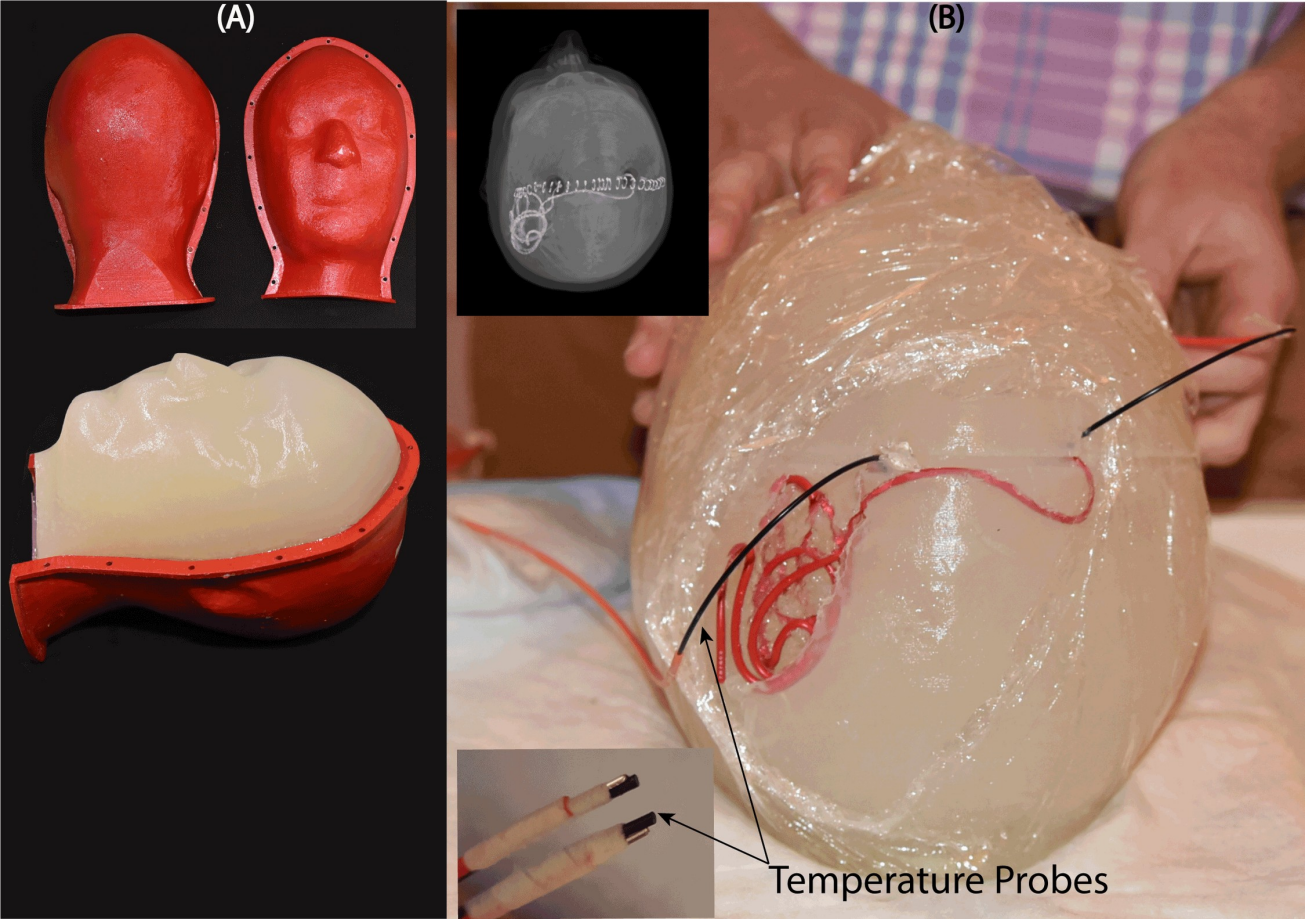
(A) Anthropomorphic head phantom based on MRI of a healthy volunteer (B) 3D printed DBS lead phantoms were used as a guide to shape wires in the form of patient-derived trajectories. Wires were implanted into semi-solid anthropomorphic head phantoms with temperature probes attached to their tips for temperature measurement during MRI with the body coil and the rotating coil system.

An anthropomorphic head phantom was designed and 3D-printed based on the structural MRI of a healthy volunteer. The mold was composed of two sagittal sections connected through a rim (Fig *5*A). Phantom dimensions were approximately 16 cm ear-to-ear and 27 cm from top of head to bottom of neck. The mold was filled with agarose-doped saline solution (5L water, 14g NaCl) through a hole at the bottom. A relatively high percentage of agarose (4%) was used which resulted in a semi-solid gel that could stand alone and support the implants. The electric properties of the gel were measured using a dielectric probe kit (85070E, Agilent Technologies, Santa Clara, CA) and a network analyzer) to be *σ* = 0.47 *S*/*m* and *ε*_*r*_ = 77. Leads were implanted into the gel following the entrance point, angle, and trajectories as observed from CT images of the patient (Fig *5*B). Fluoroptic temperature probes (OSENSA, BC, Canada) were secured at the exposed tips of the wires for temperature measurements. A third probe was inserted to the center of the head phantom for background measurements.

### RF exposure

Experiments were performed at a 1.5T Magnetom Avanto system using the rotating coils system as well as the built-in body coil for comparison. To better control the RF exposure, gradient coils were disabled and a train of 1ms rectangular RF pulses were transmitted using the “*rf_pulse*” sequence from Siemens Service Sequence directory. Scan duration was set to 120 s for both body coil and the rotating coil. The flip angle, however, was adjusted to make sure that all cases had the same level of power absorbed in the head phantom to allow for a fair comparison. The absorbed power in the phantom was determined by subtracting the power dissipated in the coil and the reflected power from the forward power in the coil (assuming negligible radiation):

$$P_{phantom}=P_{forward}-P_{reflected}-P_{coil_{loss}}$$ [1]

These values, registered as “*TALES powers*” in the scanner log file, are measured in real time at a hardware component called Transmit Antenna Level Sensor (TALES) which resides between the output of the RF amplifier and the coil. In our case, an rf_pulse sequence with TR = 6.5 ms and flip angle = 180° generated 99 W power at the head phantom when transmitted with the body coil. For the rotating coil, the TR was kept the same but flip angle was adjusted to 150° which generated 101 W power at the head phantom. Note that considering the weight of the phantom (~4kg), these values generated a global head SAR of ~25W/kg which is significantly higher than the FDA recommended a limit of 3.2 W/kg for clinical applications. Such high SAR value was necessary to produce enough heating at the tips of the lead wires to be above the noise in all experiments.

### Temperature measurements

At the start of each experiment, when the head phantom was at iso-center of the body coil, we measured the temperature rise at the tips of right and left implants by transmitting two-minute rf_pulse sequences using the body coil. Left and right leads were labeled as contralateral or ipsilateral depending on the IPG side. For patients 1–4, the IPG was planned to be implanted in the left pectoral pocket (see Fig 2, showing end segments of both leads routed toward the left side of the head). For these patients, the left lead was labeled as ipsilateral and right lead was labeled contralateral. For patients 5–10 the IPG was planned to be implanted in the right pectoral region, thus in these patients the right lead was labeled ipsilateral and left lead was labeled as contralateral. After measurements with the body coil, the phantom was left for 15 minutes to cool down. Measurements with the rotating coil started with the coil at its default position (feed up, *θ* = 0°). The rf_pulse protocol was transmitted for 2 minutes and temperatures at the tips of ipsilateral and contralateral leads as well as the background probe were recorded. The coil was then rotated to the left at 20° increments until all accessible rotation angles from 0° to 140° were covered (see Fig *2*). The coil was consequently repositioned at 0° and rotated to the other direction to cover angles from 360°-220°. Depending on the heating, the phantom was left for 15–30 minutes between each experiment to return to baseline temperature. At the end of each experiment we added a few extra measurement points around the position that produced the minimal heating on each lead to better estimate the optimum position angle.

### Measurement vs simulations

To use the rotating coil system in practice, it is necessary to be able to find the optimum rotation angle for each patient in a way that is safe and does not require pre-scan imaging with high-SAR sequences. One possible approach is to perform patient-specific simulations to estimate the vicinity of the optimum angle, and then fine-tune the positioning of the coil by minimizing the image artifact on low-SAR pre-scans that do not heat up the lead [39]. To have an estimation of how well simulations could predict measurement results, we simulated patient 10 in a rotating coil and calculated the maximum of 1g-averaged SAR around the tips of ipsilateral and contralateral leads for a range of coil rotation angles. The birdcage coil was modeled as a low-pass coil with 16 legs and two end rings constructed from 13mm copper strips (*σ* = 5.8×10^7^ *S*/*m*) and tuned to 64MHz, similar to the constructed prototype. The RF gradient shield of the scanner was modeled as an open-ended cylindrical copper sheet (radius = 35cm, length = 110cm). A 50Ω coaxial cable was designed as the feed and connected to one of the legs over a matching port. DBS lead models were reconstructed from CT images and registered in a homogeneous head phantom. Model preparation and simulation setup were same as described in our previous works [20, 28]. Briefly, DBS leads were composed of four cylindrical contacts (outer diameter = 1.27 mm, wall thickness = 150 μm), connected through a solid straight central core (diameter = 260 μm) and embedded in polyurethane insulation (diameter = 1.27 mm, σ = 10^−10^s/m, ε_r_ = 3.5 [40]). Electrode contacts were made of 90%:10% platinum-iridium (Pt:Ir, σ = 4×10^6^S/m) positioned 0.5 mm apart. The coil was then rotated around the head model with 11.25° increments (32 positions), and at each rotating angle the input power of the coil was adjusted to generate the B_1_^+^ = 2μT on an axial circular plane below electrode tips (Fig 1D). The 1g-averaged SAR was then recorded from two 2cmx2cmx2cm cubic areas surrounding ipsilateral and contralateral leads and its maximum value was registered for that rotation angle. Because we were interested in comparing the coil rotation angle corresponding to the simulated maximum SAR to the coil rotation angle corresponding to the measured maximum temperature rise (and not the absolute values of SAR or ΔT), we normalized all SAR values to the global maximum SAR value, and all ΔT values to the global maximum temperature rise.

## Results

**Fig 6:**
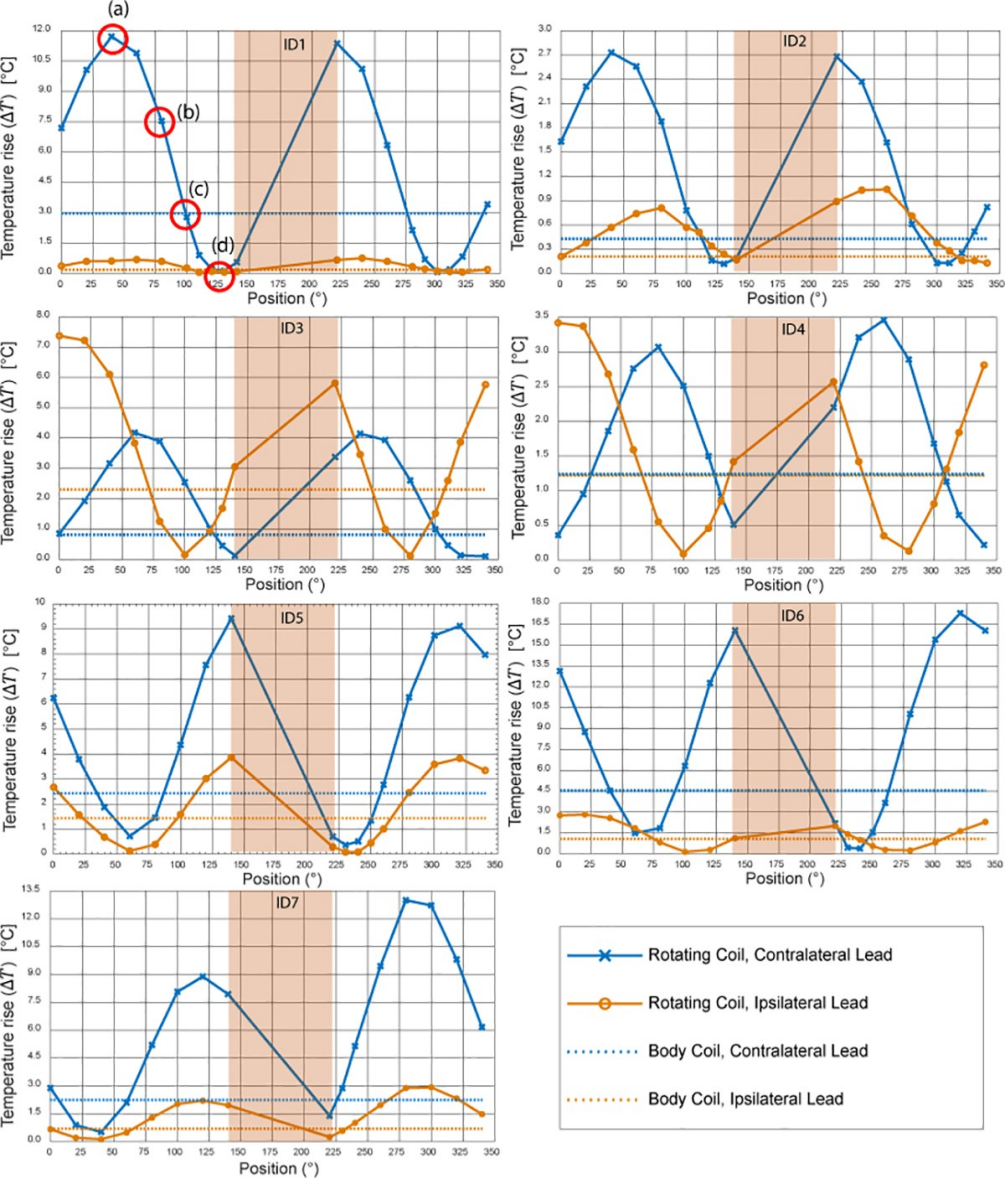
Temperature rise at the tips of ipsilateral and contralateral leads after 2 minutes of RF exposure. Red circles show coil positions corresponding to temperature curves of Fig 8.

**Fig 7:**
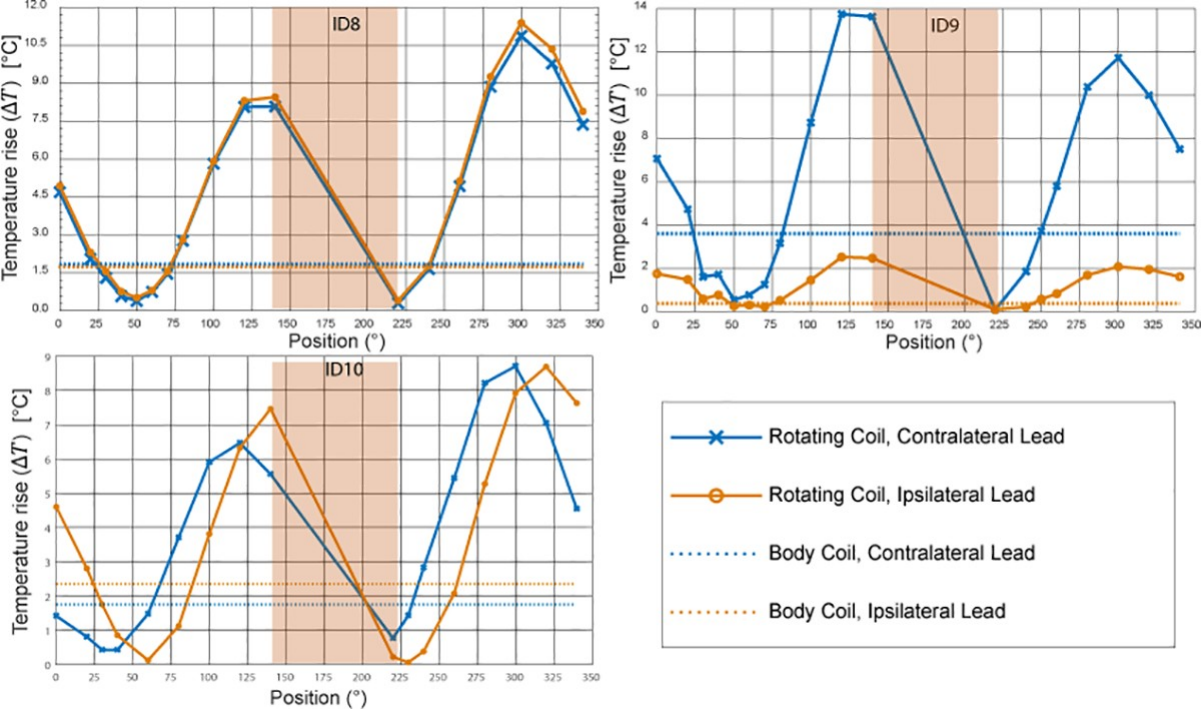
Temperature rise at the tips of ipsilateral and contralateral leads after 2 minutes of RF exposure (continued).

**Fig 8:**
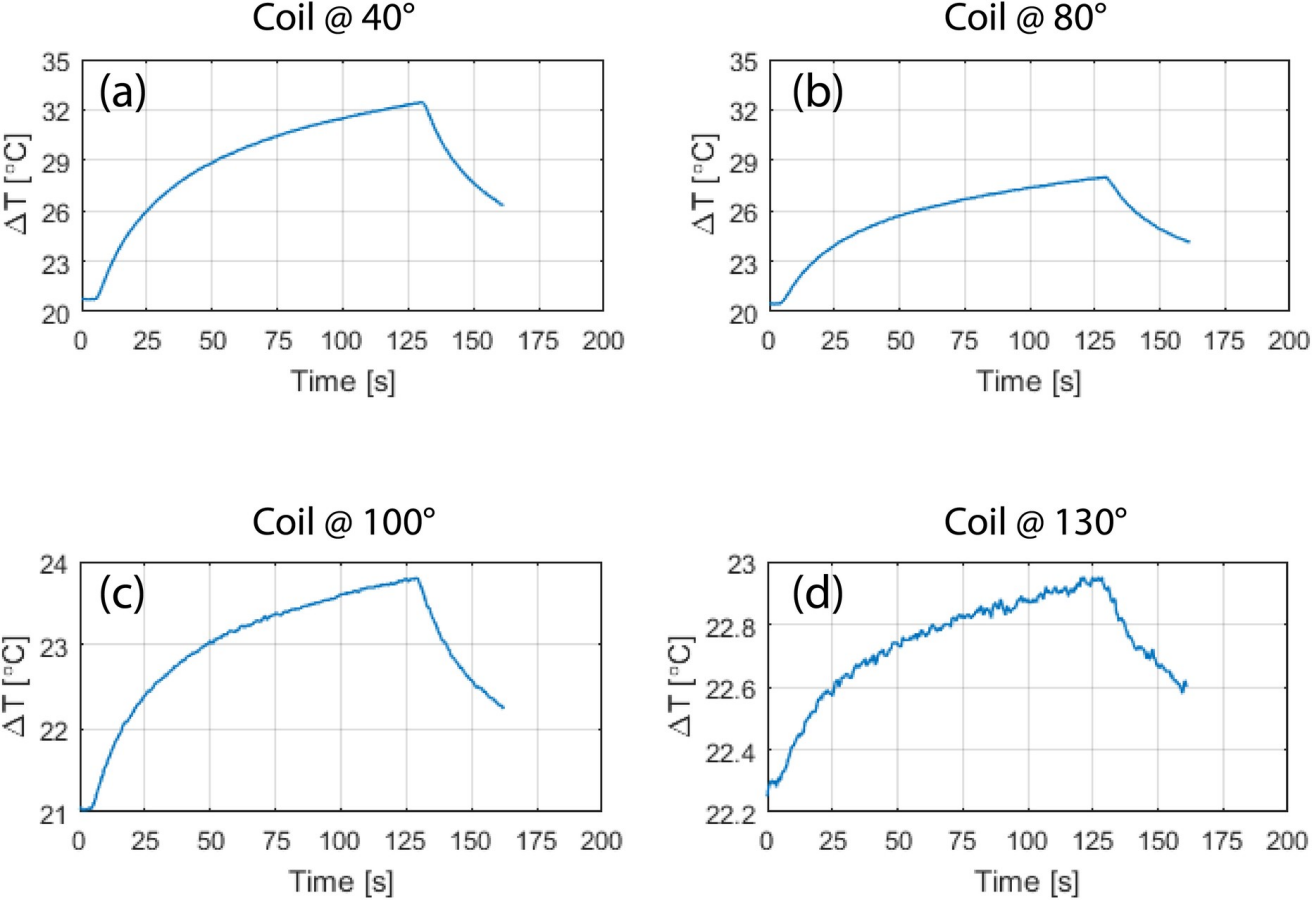
Time evolution of temperature in the tissue at the tip of contralateral lead of patient 1 for the coil in four different rotational positions corresponding to red circles annotated in Fig 6.

Fig 6 and Fig 7 show the result of temperature measurements at the tips of ipsilateral and contralateral leads of patients 1–10 for the range of accessible coil rotation angles during 2-minute RF exposure at the global SAR level of 25 W/kg. For each patient, the temperature rise at the tips of the leads are also given when the built-in body coil was used (straight dashed lines). Fig 8 gives the time evolution of temperature around the tip of the contralateral lead in patient 1 for four coil rotation angles at (a) 40°, (b) 80°, (c) 100°, and (d) 230° as an example.

To quantify the performance of the reconfigurable coil to reduce the heating of implanted leads, we defined a factor called *heat reduction percentage* or HRP for each lead and at each coil rotation angle as:

$$HRP=\frac{100(\Delta T_{cp}-\Delta T_{RB})}{\Delta T_{cp}}$$ [2]

where Δ*T*_*CP*_ is the temperature rise at the tip of the lead at the end of the 2-minute measurement with the built-in body coil and Δ*T*_*RB*_ is the temperature rise at the end of 2-minute measurement with the rotating birdcage coil. A positive HRP at a rotation angle *θ* indicates that for the same level of global SAR, the rotating birdcage coil positioned at *θ* will generate less heating at the tip of the implanted wire compared to the body coil. In contrast, a negative HRP at an angle *θ* indicates that the rotating coil positioned at *θ* generates more heating at the tip of the implanted wire compared to the body coil when both coils generate the same level of global SAR.

### Optimal rotation angle for single leads

Table 1 gives the HRP values for ipsilateral and contralateral leads of each patient at two distinct coil rotation angles which minimized either the heating of ipsilateral lead or the heating of contralateral lead. Table entries highlighted green show the lead for which the heating was minimized. For example, in patient 9, coil at *θ* = 230° minimized the heating of ipsilateral lead (HRP_Ipsi_ = 97) and coil at *θ* = 40° minimized the heating of contralateral lead (HRP_contra_ = 63). As it can be observed from Table 1 (as well as Figs 6 and 7), for each of the 20 leads there existed an optimum coil rotation angle that reduced the heating of the lead to a level well below the heating produced by the body coil. On average, a substantial heat reduction of 80%±19% was achieved for single leads. In 33% of cases (patients 5,7 and 8) the optimum rotation angles that minimized the heating of ipsilateral and contralateral leads were the same. For the rest of patients, the optimal angles for ipsilateral and contralateral leads were different and in 40% of cases (patients 2,3,4, and 10) the rotation angle that minimized the heating at one lead increased the heating at the other one.

**Table 1:** HPR values for ipsilateral and contralateral leads of patients 1–10.

		*HRP* = 100(Δ*T*_*CP*_−Δ*T*_*RB*_)/Δ*T*_*CP*_	
Patients	Coil angle	Ipsilateral Lead	Contralateral Lead
ID1	130°	61	96
	300°	33	97
ID2	340°	19	-91
	300°	-81	70
ID3	280°	95	-218
	340°	-149	87
ID4	100°	93	-104
	340°	-128	82
ID5	230°	93	83
ID6	100°	87	-39
	240°	7	90
ID7	40°	82	77
ID8	220°	77	84
ID9	230°	97	19
	40°	75	63
ID10	250°	95	-73
	40°	-60	74

For each patient, HPR is given for rotating angles that minimized the heating either at ipsilateral or contralateral lead.

### Optimal rotation angle for double leads

From the results of the previous section one can see that for most realistic bilateral DBS implants there is not an optimal coil rotation angle that maximally reduces the heating of both ipsilateral and contralateral leads. For all patients however, it was possible to find an intermediate coil position that reduced the heating of both leads to a level below the heating produced by the body coil, albeit this position was not always optimum for each lead alone. Table 2 shows the rotation angle that maximized the value of HRP_total_ = HRP_ipsi_+HRP_contra_. As it can be observed from the table, for all ten patients we were able to reduce the heating of both ipsilateral and contralateral leads to a level below the heating produced by the body coil. When optimized for bilateral leads, an average heat reduction factor of 65%±25% was achieved.

**Table 2:** HPR values for ipsilateral and contralateral leads of patients 1–10.

		HRP = 100(Δ*T*_*CP*_−Δ*T*_*RB*_)/Δ*T*_*CP*_	
Patients	Coil angle	Ipsilateral Lead	Contralateral Lead
ID1	130°	61	96
ID2	140°	19	56
ID3	130°	27	44
ID4	130°	63	44
ID5	230°	93	83
ID6	250°	48	66
ID7	40°	82	77
ID8	220°	77	84
ID9	220°	72	97
ID10	220°	24	86

For each patient, HPR values are given for the rotating angle that maximized HRP_ipsi_+HRP_contra_.

### Simulations as a mean to estimate coil position

**Fig 9:**
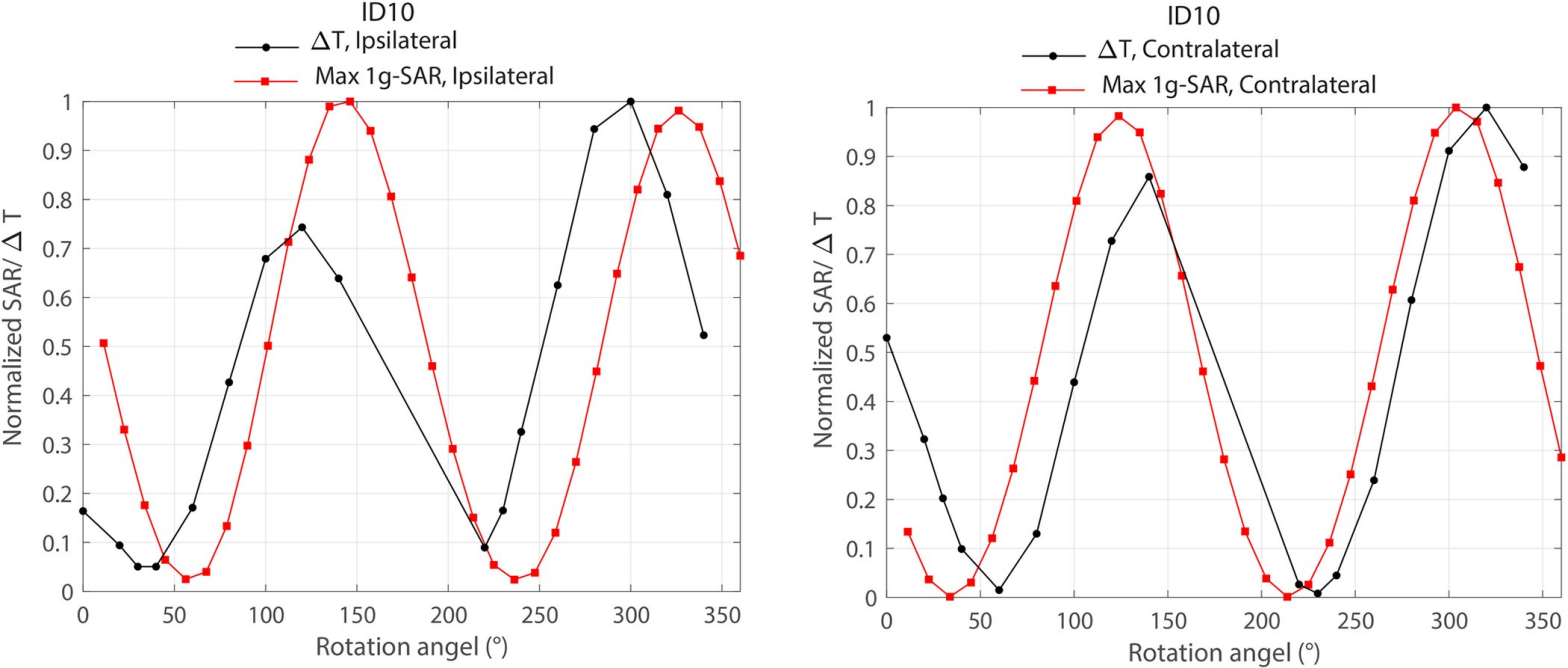
Comparison of simulated maximum 1g-averaged SAR and measured temperature rise around ipsilateral and contralateral leads of patient 10. SAR and temperature values were normalized to their corresponding maximum before overlaying the plots.

Fig 9 shows the overlaid results of local SAR and temperature rise around ipsilateral and contralateral leads of patient 10 as a function of coil rotation angle. As it can be observed from the Fig, simulations could estimate the optimum rotation angle within 20° of accuracy. It is important to note however that another level of uncertainty was introduced by performing simulations on the models that were based on the original CT images of the patient, rather than on the models constructed from CT images of implemented leads in the head phantom used in measurement. Although for measurements we tried our best to implant the wires in the gel phantom following visual guidance from patient’s CT images, we could not replicate the exact positioning of the lead as happened in the patient. Numerical models on the other hand, represented the exact lead orientation of the patient which explains the slight discrepancy between simulations and measurement results.

## Discussion and conclusion

This work presents experimental results of temperature measurements at the tips of bilateral DBS leads during MRI using a new rotating coil system that can be adjusted for each patient to reduce the local heating of the implant. The idea of generating and steering electric-field free regions in MR transmit coils to reduce implant heating was originally introduced by Atalar’s group [25, 32] and later adopted in studies that designed implant-friendly modes in parallel transmit volume coils at 3T [23, 24, 26, 41]. Techniques based on tailoring the transmit pulse usually adopt a cumbersome simulation approach to determine the magnitude and phase of the signal at each transmit channel to shape the electric field in the region of interest (i.e., around the implant) while maintaining a user-defined threshold of B_1_ homogeneity throughout the sample. Although using a multi-channel transmit methodology allows more degrees of freedom for field tailoring, and thus could potentially achieve a better SAR reduction for complicated implant trajectories (e.g., multiple leads), such benefit comes with the drawback of rendering the technique complicated for application in clinic. The rotating coil system introduced here has the advantage of having a very simple setup, however such ease of operation comes at the expense of lack of control on the shape of the low electric field region. In other words, apart from *“steering”* the low E-field region by rotating the coil around patient’s head, there is no other control on the shape and extend of this low field region. It is established that the SAR amplification at the tips of elongated implants depends on the coupling between the tangential component of the incident electric field and implanted wires [34, 35, 42]. As implanted DBS leads have complex trajectories consisting of segments that cannot be contained in one plane (see Fig *3*), it is important to assess the performance of any SAR-reducing strategy in models based on real patient data. This work presents the first report of such assessment, demonstrating promising results in possibility of reducing the SAR at the tips of both left and right DBS leads to levels below the SAR generated by standard body coils. It should be noted however, that the current study is limited to the assessment of heating at the tips of DBS leads in isolation, i.e., prior to their connection to the extension cables and the implanted pulse generator; therefore, the results presented here should not be the extended to other configurations. Further investigation is necessary to establish the efficacy of the technique in a fully implanted system.

From Table 1 it can be observed that for patients 5, 7 and 8 the optimum coil rotation angle that minimized the SAR at the tips of right and left leads was the same. A closer look at the lead trajectory in these patients (Fig 1) reveals that ipsilateral and contralateral lead trajectories are routed such that their trajectories are substantially parallel in this case. We recently showed that it is possible to implement simulation-driven instructions in the implantation of extracranial portion of DBS leads during the surgery to reduce the SAR, without requiring additional equipment or adding to the surgical time [43]. Therefore, surgeons can be instructed to subcutaneously rout the leads such that their trajectories be maximally parallel. This will allow the reconfigurable coil system to perform as efficiently in the case of patients with bilateral leads as it does for unilateral implants. Instructing surgeons to follow a lead management strategy can also reduce the uncertainty in the overall shape of extracranial trajectory of the lead. Unlike the meticulously planned intracranial lead trajectory, for which almost every neurosurgeon follows textbook guidelines to choose the entry point on the skull and the angle of penetration to the target nuclei, there are no rules or guidelines for the placement of extra cranial portions of the leads. As a result, there is a substantial patient-to-patient variation in the trajectory of extracranial portion of the leads with many cases exhibiting abrupt changes in the orientation of lead segments. This increases the sensitivity of coil positioning, as small deviations from the optimum angle could cause parts of the lead to fall out of low electric field region.

Our preliminary results comparing simulation results and measurements indicated that patient-specific models are a viable choice to predict the whereabout of the optimum coil angle. Once this initial angle is predicted, the position can be fine-tuned by minimizing the image artifact using low-SAR pre-scans that do not heat up the lead. More work is needed however, to quantify the agreement of numerical models with measurements and to devise detailed strategies to fine tune the coil position for each patient.
